# Most Japanese individuals are genetically predisposed to recognize an immunogenic protein fragment shared between COVID-19 and common cold coronaviruses

**DOI:** 10.12688/f1000research.51479.1

**Published:** 2021-03-10

**Authors:** Johannes M. Dijkstra, Aaron P. Frenette, Brian Dixon

**Affiliations:** 1 Institute for Comprehensive Medical Science, Fujita Health Universit, Toyoake-shi, 470-1192, Japan; 2 Department of Biology, University of Waterlo, Waterloo, ON, N2L 3G1, Canada

**Keywords:** COVID-19, T cell, MHC, HLA, peptide, epitope, Japanese, VYIGDPAQL, SPRWYFYYL

## Abstract

In the spring of 2020, we and others hypothesized that T cells in COVID-19 patients may recognize identical protein fragments shared between the coronaviruses of the common cold and COVID-19 and thereby confer cross-virus immune memory. Here, we look at this issue by screening studies that, since that time, have experimentally addressed COVID-19 associated T cell specificities. Currently, the identical T cell epitope shared between COVID-19 and common cold coronaviruses most convincingly identified as immunogenic is the CD8 ^+^ T cell epitope VYIGDPAQL if presented by the MHC class I allele HLA-A*24:02. The HLA-A*24:02 allele is found in the majority of Japanese individuals and several indigenous populations in Asia, Oceania, and the Americas. In combination with histories of common cold infections, HLA-A*24:02 may affect their protection from COVID-19.

## Introduction

**Figure 1.**
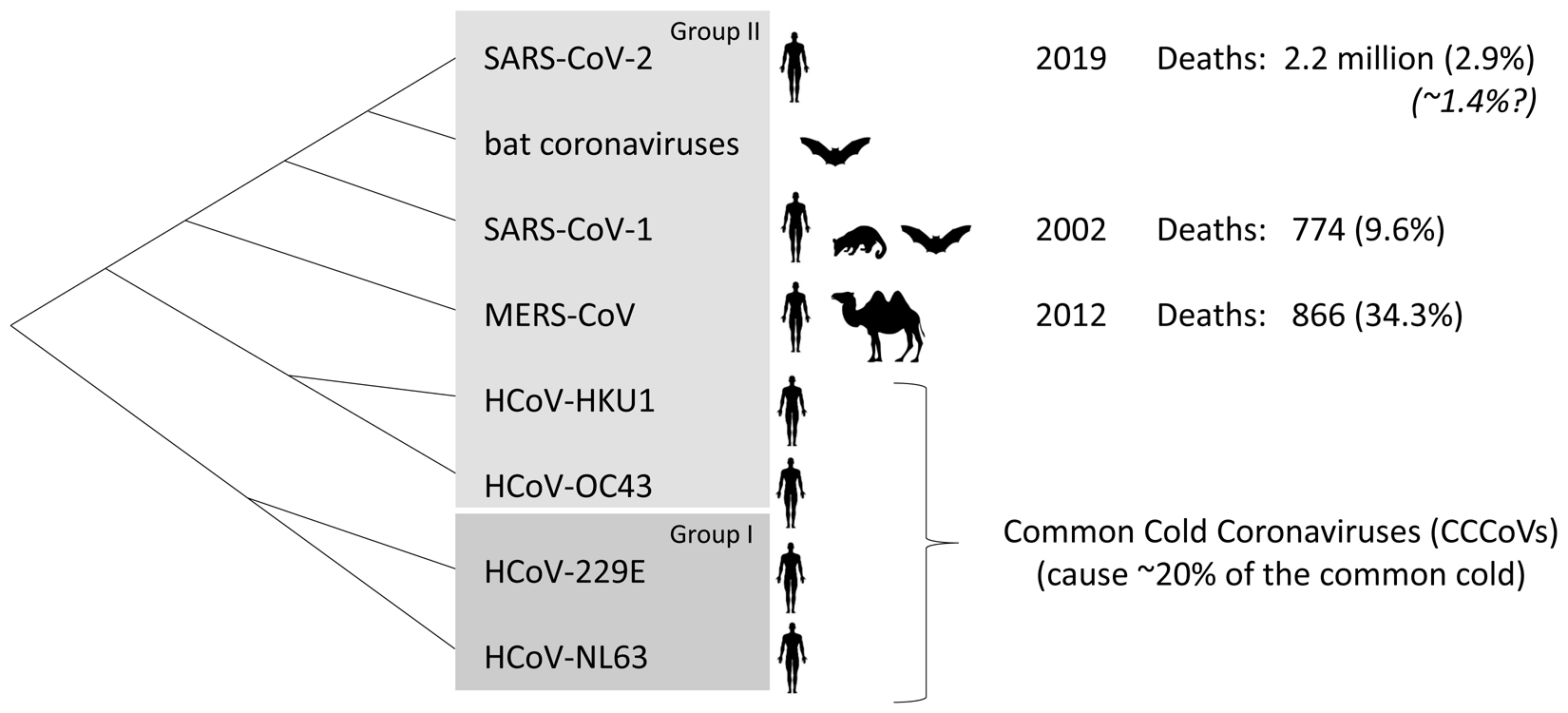
Cladogram of the phylogeny of coronaviruses infecting humans ( Ceraolo & Giorgi, 2020; Forni *et al.*, 2017). Viruses closely related to SARS-CoV-2 are found in bats. Bats and civets are the probable sources of SARS-CoV-1, and camels are alternative hosts for MERS-CoV. The first reported outbreaks in people by infections with SARS-CoV-1, MERS-CoV, and SARS-CoV-2 occurred in 2002, 2012, and 2019, respectively, with differences in number of deaths and case fatality ratios among registered cases (percentages indicated in regular font between parentheses) ( https://www.who.int/publications/m/item/summary-of-probable-sars-cases-with-onset-of-illness-from-1-november-2002-to-31-july-2003; https://www.who.int/health-topics/middle-east-respiratory-syndrome-coronavirus-mers; https://coronavirus.jhu.edu/map.html). SARS-CoV-2 infections and deaths are not always registered and based on data from New York it was estimated that the true fatality rates may be ~1.4% (Italic font) ( Yang *et al.*, 2021).

The virus causing the COVID-19 pandemic is severe acute respiratory syndrome coronavirus 2 (SARS-CoV-2) ( Wu *et al.*, 2020; Zhou *et al.*, 2020). SARS-CoV-2 is one of the seven coronaviruses that are known to infect humans, the others being SARS-CoV-1 (causing SARS), Middle East respiratory syndrome coronavirus (MERS-CoV), and the common cold coronaviruses (CCCoVs): human coronavirus OC43 (HCoV-OC43), HCoV-HKU1, HCoV-229E, and HCoV-NL63. These coronaviruses belong to the serological/phylogenetic clades designated as group I (alphacoronaviruses) and group II (betacoronaviruses) ( Figure 1). SARS-CoV-1 and MERS-CoV infected relatively few people and therefore should have little effect on global cross-virus immune memory. On the other hand, the CCCoVs cause ~20% of common cold cases, are globally distributed, and all adults have probably been infected with them multiple times in their lives ( Hirsch *et al.*, 2013; Mäkelä *et al.*, 1998; Zhou *et al.*, 2013).

The immune defense against viruses includes both innate and adaptive immune responses. Cell types that specialize in adaptive immunity (immune memory) are B cells, CD4 ^+^ T cells, and CD8 ^+^ T cells. B cells can secrete antibodies, but there is probably little or no protective cross-virus anti-SARS-CoV-2 humoral immunity deriving from infections by CCCoVs (e.g., Amanat *et al.*, 2020; Shrock *et al.*, 2020). CD8 ^+^ T cells recognize peptides presented by major histocompatibility complex class I (MHC-I) cells and can kill the presenting cell; the peptides bound by MHC-I are approximately 8-13 amino acids (aa) length, and mostly are 9 aa (“9-mers”) ( Rammensee *et al.*, 1995; Schellens *et al.*, 2015). CD4 ^+^ T cells recognize peptides if presented by MHC class II (MHC-II) molecules and help regulate immune responses; the peptides bound to MHC-II typically are 12-25 aa ( Rammensee *et al.*, 1995), although the part binding within the MHC-II groove is only 9 aa as commonly found for MHC-I ( Stern & Wiley, 1994). In the case of MHC-I alleles, available computational software provides a powerful in silico tool in predicting which peptides are presented. For a broader discussion on T cell functions in COVID-19 patients, including whether T cell responses are always beneficial or could also be detrimental, we refer to other studies ( Altmann & Boyton, 2020; Bacher *et al.*, 2020b; Jarjour *et al.*, 2020).

In the spring of 2020, Nguyen *et al.* (2020) and Dijkstra & Hashimoto (2020) reported on possible MHC-I binding epitopes shared between CCCoVs and SARS-CoV-2 based on in silico analyses and speculated on cross-virus T cell immune memory. Whereas Nguyen *et al.* (2020) made a comprehensive analysis of possible MHC epitopes of SARS-CoV-2, Dijkstra & Hashimoto (2020) concentrated on identical 9-mers shared between SARS-CoV-2 and at least one of the CCCoVs. Probably for that reason, Dijkstra & Hashimoto (2020) were more accurate in identifying such 9-mers, as they found >230 whereas Nguyen *et al.* (2020) only listed 144. These identical 9-mers, or even 8-mers, were mostly found in several well-conserved nonstructural proteins that are expressed as part of the ORF1ab polyprotein, while they are absent or nearly absent in the structural proteins S, M, N, and E ( Dijkstra & Hashimoto, 2020; Lee *et al.*, 2020; Nguyen *et al.*, 2020). Since then, it has been shown indeed that a recent history of CCCoV infections reduces the severity of COVID-19 infections ( Sagar *et al.*, 2021), and a considerable number of studies have investigated SARS-CoV-2 T cell epitopes (summarized in this article). The present study screened the recent literature to investigate—in line with the hypothesis of our earlier report ( Dijkstra & Hashimoto, 2020)—which of the identical peptides shared between SARS-CoV-2 and any of the CCCoVs have been confirmed experimentally to bind to MHC molecules and/or to stimulate T cells. Arguably, the only one of such peptides convincingly reported as immunogenic by independent research groups is the helicase-derived peptide VYIGDPAQL which binds MHC-I allele HLA-A*24:02 and then can stimulate CD8 ^+^ T cells. In some populations, such as the Japanese, HLA-A*24:02 is found in >50% of the individuals and the allele may affect their resistance against COVID-19.

## Methods

As we did before ( Dijkstra & Hashimoto, 2020), proteins encoded by a reported genomic sequence for SARS-CoV-2 (GenBank MN908947; Wu *et al.*, 2020) were compared with those for HCoV-OC43 (NC_005147; Vijgen *et al.*, 2005), HCoV-HKU1 (NC_006577; Woo *et al.*, 2005), HCoV-229E (NC_002645; Thiel *et al.*, 2001), and HCoV-NL63 (NC_005831; van der Hoek *et al.*, 2004) by performing BLAST homology searches at the NCBI database ( https://blast.ncbi.nlm.nih.gov/Blast.cgi) and by making multiple sequence alignments using CLUSTALW software ( https://www.genome.jp/tools-bin/clustalw); continuous stretches of 9 aa acids identical between SARS-CoV-2 and one of the other viruses were identified manually. A complete list of the detected 9-mers, minus a very few that we had missed at that time, are shown in Dijkstra & Hashimoto (2020). Furthermore, from published reports found by Google and PubMed searches, sequences of SARS-CoV-2 peptides reported to activate T cells were compared with the above listed CCCoV proteomes using tblastn (align) function at the NCBI database, in search for identical sequences in the case of 8-mers (which we did not find) or stretches of ≥9 consecutive identical aa in the case of 9-mers or larger peptides. The peptide sequences collected by either method were screened against the Immune Epitope Database (IEDB; http://www.iedb.org/; Dhanda *et al.*, 2019) for reports in the human species context, and, unless these database reports represented the same studies that also appeared in article form, their IEDB information was added to Table 2 (available here). The collected peptide sequences were also analyzed by ANN 4.0 software at IEDB Analysis Resource ( http://tools.immuneepitope.org/mhci/) for prediction of their affinity to a set of representative human MHC-I alleles, which were chosen because of their global abundancy, their relevance for the presented data, or as representatives of MHC-I supertypes ( Lund *et al.*, 2004). Identical 9-mers for which no MHC binding was found or predicted, for which no labeling or activation of T cells was reported, and which were not part of larger immunogenic T cell epitopes, were not included in Table 2.

The HLA allele frequencies as shown in Table 3 are based on data as summarized in the Allele Frequency Net Database http://www.allelefrequencies.net/ (settings: HLA > HLA classical allele freq search) ( Gonzalez-Galarza *et al.*, 2020).

## Results and discussion

### The first two experimental studies on CCCoV-derived anti-SARS-CoV-2 cross-virus T cell memory

**Figure 2.**
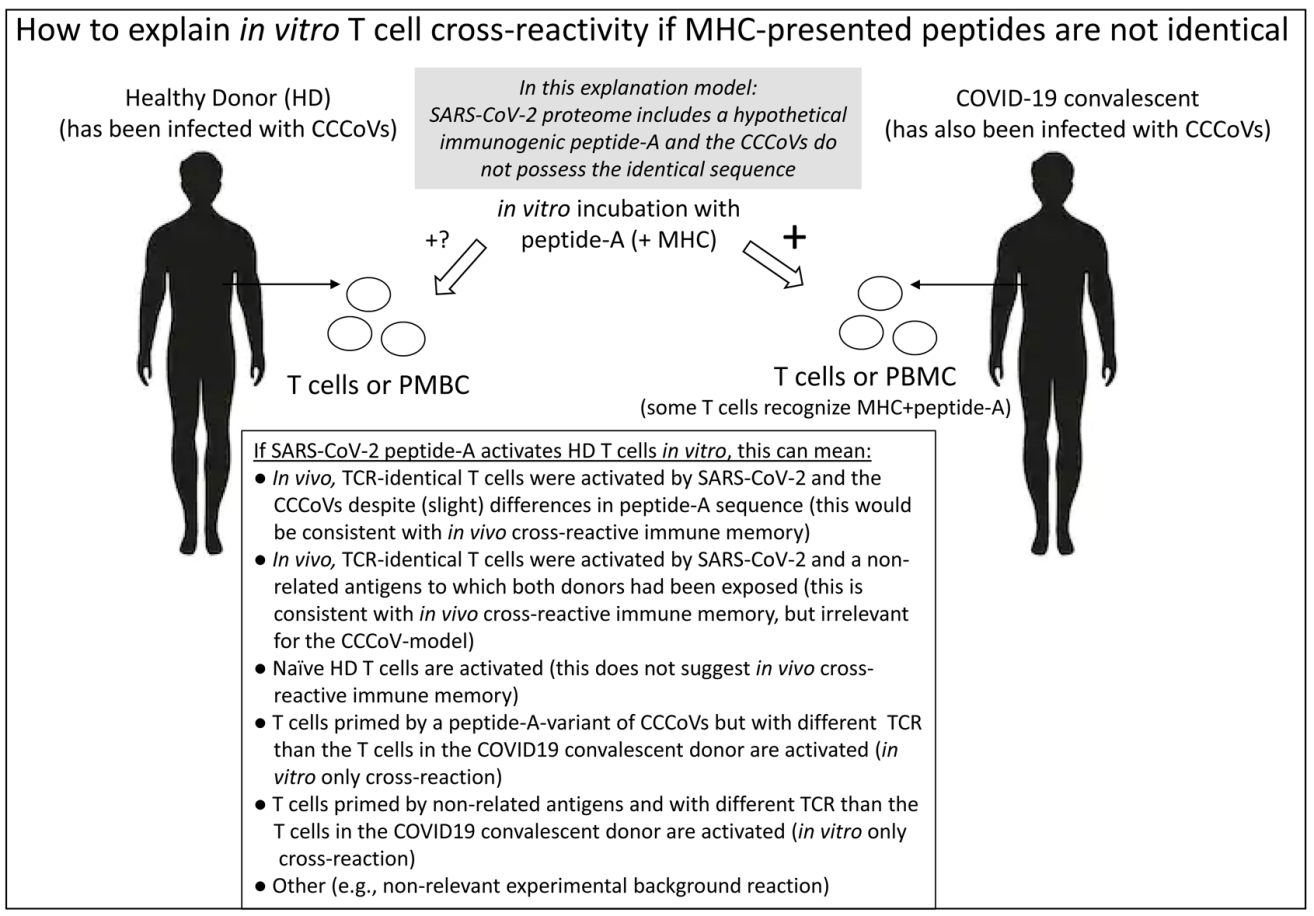
Theoretical possibilities for explaining *in vitro* activation of T cells from healthy donors (HD) by a hypothetical immunogenic SARS-CoV-2 peptide (“peptide-A”) that has no perfect sequence match in the CCCoVs. Even if the CCCoVs do possess a very similar sequence, the *in vitro* activation does not need to be indicative for peptide-A being an epitope for *in vivo* cross-virus T cell memory. In the model, peptide-A is either used directly or as part of pMHC complexes, and the T cells are stimulated after their isolation or as part of PBMC.

The first two studies that experimentally investigated possible CCCoV-induced T-cell memory against SARS-CoV-2 were Grifoni *et al.* (2020) and Braun *et al.* (2020) ( Leslie, 2020). Braun *et al.* (2020) tested CD4 ^+^ T cell activation using S (spike) protein derived peptide pools, whereas Grifoni *et al.* (2020) investigated both CD4 ^+^ T and CD8 ^+^ T cell activations using peptide pools derived from various SARS-CoV-2 proteins. Both groups found SARS-CoV-2 peptide pools to activate T cells from healthy donors (HD), proposedly representing memory T cells primed by similar peptides during earlier CCCoV infections. Notably, T cells of HD were also activated if their peripheral blood mononuclear cells (PBMC) were incubated with pools of peptides derived from not very well conserved SARS-CoV-2 proteins such as S ( Braun *et al.*, 2020; Grifoni *et al.*, 2020), although—depending on the virus isolates—SARS-CoV-2 S protein does not share identical 9-mers with any of the CCCoVs but may share two identical 8-mer sequences ( Dijkstra & Hashimoto, 2020). Braun *et al.* (2020) assumed that even a <50% identity between the corresponding SARS-CoV-2 and CCCoV peptides might explain the assumed cross-virus CD4 ^+^ T cell memory. Similarly, the authors of the Grifoni *et al.* (2020) study—answering our question on how S-derived peptides could activate cross-virus CD4 ^+^ and CD8 ^+^ T cell memory despite not sharing identical 9-mers or (presumably) immunogenic identical 8-mers—explained that CD8 ^+^ T cell activation in their type of *in vitro* assay could be found for a substantial part of any peptides sharing only ≥70% identity and that for CD4 ^+^ T cell activation the requirements for peptide similarity were even lower (see comments section below the Grifoni *et al.*, 2020 article at https://www.cell.com/cell/fulltext/S0092-8674(20)30610-3). Although it has been well established that T cells can be promiscuous in recognizing pMHC (MHC + peptide) complexes, the extents to which this is relevant for *in vivo* T cell memory and does or does not tend to involve peptides with very similar sequences are being debated (e.g., Grant *et al.*, 2016; Petrova *et al.*, 2012). As explained by Peng *et al.* (2020), *in vitro* observations that suggested the existence of CCCoV-primed anti-SARS-CoV-2 “cross-reactive” T cell memory might alternatively be caused by the activation of naive T cells or of T cells primed by not-so-similar peptides of non-related pathogens. Possibilities for explaining *in vitro* observations suggesting cross-virus T cell memory in the case of non-identical peptides are summarized in Figure 2. Naturally, the proportions of false negative and false positive outcomes depend on the sensitivity of the assay. The best chance for *in vitro* results truly representing stimulation of the same T cells (TCR-identical T cells) activated during CCCoV and subsequent SARS-CoV-2 infections—hence representing functional memory—is if the MHC-presented peptides are identical. Hence, the present article predominantly focuses on such identical peptides.

### Summary, from the perspective of possible cross-virus immune memory, of studies that identified SARS-CoV-2 T cell epitopes

Table 1 summarizes the Grifoni *et al.* (2020) and Braun *et al.* (2020) studies, as well as later studies that experimentally investigated potential SARS-CoV-2 T cell epitopes. Section A of the table summarizes studies that only investigated peptide pools and/or intact proteins, and section B summarizes the studies that (also) investigated individual peptides. The table explains whether indications for CD4 ^+^ and/or CD8 ^+^ T cell activation were observed, whether MHC association was addressed and/or MHC binding tested, and whether indications for SARS-CoV-2 and CCCoV cross-virus T cell memory were investigated and observed. Importantly, the consensus was that anti-SARS-CoV-2 T cell memory in individuals that had only been infected with CCCoVs was weak and only detectable in a subset of individuals ( Sette & Crotty, 2020). It should be noted that none of the studies listed in Table 1 had specifically selected individuals with a known recent history of CCCoV infection, although presumably in such group a stronger T cell response against SARS-CoV-2 antigens would be found ( Sagar *et al.*, 2021).

**Table 1.** Summary of experimental studies on SARS-CoV-2 proteins/peptides in relation to T cell activation.

(A) Studies that only used peptide pools or intact proteins				
	Indications for	Indications for		
	SARS-CoV-2-	cross-virus	MHC	
Reference	specific T cells ^a^	T cell memory ^b^	alleles ^c^	The investigated peptides (and positive findings for cross-virus shared 9-mer sequences) ^d, e^
Anft *et al.*, 2020	CD4, CD8	CD4, CD8	n.d.	peptide pools derived from S
Bacher *et al.*, 2020a	CD4	CD4	n.d.	peptides pools derived from S, M, N, E, NS6, NS7a, NS7b, NS8, ORF3a, ORF9B, ORF10, and ORF14
Braun *et al.*, 2020	CD4	CD4	n.d.	peptide pools derived from S
Dan *et al.*, 2021	CD4, CD8	n.d.	n.d.	peptide pools derived from throughout the SARS-CoV-2 proteome
Grifoni *et al.*, 2020	CD4, CD8	CD4, CD8	n.d.	peptide pools derived from throughout the SARS-CoV-2 proteome
Meckiff *et al.*, 2020	CD4	CD4	n.d.	peptide pools derived from S and M
Ni *et al.*, 2020	Yes, not specified	Yes	n.d.	S, N, and NSP5 proteins
Rydyznski Moderbacher *et al.*, 2020	CD4, CD8	CD4, CD8	n.d.	peptide pools derived from throughout the SARS-CoV-2 proteome
Steiner *et al.*, 2020	CD4, CD8	CD4, CD8	n.d.	peptide pools derived from S and N
Thieme *et al.*, 2020	CD4, CD8	CD4, CD8	n.d.	peptide pools derived from S, M, and N
Weiskopf *et al.*, 2020	CD4, CD8	CD4, CD8	n.d.	peptide pools derived from throughout the SARS-CoV-2 proteome
**(B) Studies that (also) investigated individual peptides**				
Ferretti *et al.*, 2020	CD8	CD8	a, b	peptides derived from throughout the SARS-CoV-2 proteome; activation of CD8+ T cells by **VYI** and **SPR** peptides
Gangaev *et al.*, 2020	CD8	CD8	a, b	peptides throughout the SARS-CoV-2 proteome but the preprint does not provide all details
Habel *et al.*, 2020	CD4, CD8	maybe	a, b	peptides derived from S, M, N, NSP3, NSP4, NSP6, and NSP12
Kared *et al.*, 2020	CD8	No	a, b	peptides derived from throughout the SARS-CoV-2 proteome; activation of CD8+ T cells by **VYI** and **SPR** peptides
Keller *et al.*, 2020	CD4, CD8	Yes, not specified	a	peptides derived from S, M, N, and E
Le Bert *et al.*, 2020	CD4, CD8	CD4, CD8	a	peptides derived from N, NSP7, and NSP13; activation of CD4+ T cells by **SPR** encompassing peptide
Mateus *et al.*, 2020	CD4, CD8	CD4, CD8	n.d.	peptides derived from throughout the SARS-CoV-2 proteome; activation of CD4+ T cells by LKS, YLR (+ LRK, RKH), IER (+ ERF, RFV, FVS, VSL), and NVN (+ VNR, NRF, RFN, FNV) encompassing peptides
Nelde *et al.*, 2021	CD4, CD8	CD4, CD8	a	peptides derived from throughout the SARS-CoV-2 proteome; activation of PBMC (probably CD8+ T cells) by **VYI** peptide; activation of CD4+ T cells by peptide that partially overlaps **SPR** peptide
Peng *et al.*, 2020	CD4, CD8	No	a, b	peptides derived from S, M, N, E, ORF3a, ORF6, ORF7a, and ORF8; binding of CD8+ T cells by HLA-B*07:02/ **SPR** pentamers; activation of CD4+ and CD8+ T cells by peptide encompassing **SPR** peptide
Poluektov *et al.*, 2020	n.d.	n.d.	b	peptides derived from throughout the SARS-CoV-2 proteome
Prachar *et al.*, 2020	n.d.	n.d.	b	peptides derived from throughout the SARS-CoV-2 proteome; SLA peptide bound to HLA-A*02:01; KYT, AYA, and **VYI** peptide bound to HLA-A*24:02; HRF peptide bound to HLA-B*40:01; peptide encompassing RFY (+ FYR, YRL, RLA) bound to HLA-DR4
Schulien *et al.*, 2021	CD8	CD8	a, b	peptides derived from throughout the SARS-CoV-2 proteome; activation of CD8+ T cells by **SPR** peptide; binding of CD8+ T cells by HLA-B*07:02/ **SPR** tetramers
Sekine *et al.*, 2020	CD4, CD8	CD4, CD8	(a?), b	peptides derived from S, M, N, E, ORF3a, and ORF6; activation of CD8+ T cells by **SPR** peptide
Shomuradova *et al.*, 2020	CD4, CD8	CD4, CD8	a, b	peptides derived from S, M, and N
Snyder *et al.*, 2020	CD8	n.d.	a	peptides derived from throughout the SARS-CoV-2 proteome; activation of CD8+ T cells by FVD, RIL, AIM, and IVD peptides and by a combined set of **SPR** peptide plus an SPR-overlapping peptide
Takagi & Matsui, 2020	CD8 (in HLA-A*02+ mice)	n.d.	a, b	peptides derived from NSP1-to-10
Tarke *et al.*, 2020	CD4, CD8	CD4, CD8	a, b	peptides derived from throughout the SARS-CoV-2 proteome; activation of CD4 ^+^ T cells by peptides that encompass or partially overlap LKS, YPK (+ PKC), RFY (+ FYR, RLA, LAN), FNI (+ NIC, ICQ), IER (+ ERF, RFV, FVS, VSL, SLA), **SPR** (+ PRW, RWY, WYF, YFY), or RAK (+AKH); activation of CD8 ^+^ T cells by QTV encompassing 10-mer, YAI (+AIS) encompassing 10-mer, DLT encompassing 12-mer, and by 10-13 mers that encompassed DYV, YVY, VYL, YLP, LPY, and/or PYP
Woldemeskel *et al.*, 2020	CD4 (presumably)	CD4 (presumably)	(a?)	peptides derived from S, M, and N

(a) In most of the listed studies experimental evidence was obtained for the existence of SARS-CoV-2-specific CD4 ^+^ and/or CD8 ^+^ T cells in COVID-19 convalescent donors

(b) In many of the listed studies experimental evidence was obtained suggesting that CCCoV infections induced, or could induce, anti-SARS-CoV-2 T cell memory. Naturally, no samples were used of healthy donors without CCCoV infection history, and for this table, as done in the majority of the listed studies, all positive reactions in healthy donors that indicated SARS-CoV-2-specific T cell activation were interpreted as indications for possible cross-virus T cell memory. In the Habel *et al.* (2020) study, for T cells from healthy donors activations of similar extent were found for SARS-CoV-2 peptides and peptides from other pathogens for which the donors did not have an infection history.

(c) Some of the listed studies determined the association (a) of T cell responses with MHC alleles or found binding (b) of peptides to MHC alleles

(d) This column lists the proteins or peptides that were investigated. In most cases, though not all, there had been a preselection of peptides based on software predictions for MHC binding. In addition, positive findings for identical 9-mers shared between SARS-CoV-2 and at least one of the CCCoVs are summarized, with VYI and SPR peptides highlighted in bold. (e) The 3-letter names for peptides here only refer to the 9-mers "Not specified" indicates that it was not determined whether reacting cells were CD4+ or CD8+ T cells. A question mark is added if we are uncertain about what the authors did. n.d. = not determined

**Table 3.** Frequency of HLA-A*24:02 in different populations.

	% of individuals that have the allele	Allele frequency	Sample size
Taiwan Paiwan	96	0.86	51
Taiwan Tsou	98	0.78	51
Taiwan Rukai	96	0.76	50
Papua New Guinea Eastern Highlands Goroka Asaro		0.74	57
Papua New Guinea Karimui Plateau Pawaia		0.74	80
Taiwan Puyuma	88	0.64	50
Taiwan Ami	85	0.63	98
Papua New Guinea Wanigela Keapara		0.63	66
Taiwan Atayal	82	0.62	106
Ecuador Cayapa		0.61	183
New Caledonia		0.61	65
Venezuela Perja Mountain Bari		0.60	55
Taiwan Thao	90	0.60	30
Colombia Waunana NA-DHS_20	85	0.60	20
Taiwan Bunun	84	0.58	101
USA Alaska Yupik		0.58	252
Taiwan Saisiat	86	0.57	51
Taiwan Tao	78	0.54	50
Colombia Embera NA-DHS_19	93	0.54	14
Colombia/Brazil Ticuna Tarapaca NA-DHS_22	74	0.53	19
Papua New Guinea Wosera Abelam		0.51	131
Colombia/Brazil Ticuna Arara NA-DHS_21	67	0.50	17
Taiwan Siraya	78	0.47	51
Colombia North Chimila Amerindians		0.46	47
Taiwan Taroko	73	0.45	55
Colombia Arhuaco NA-DHS_16	65	0.44	17
Colombia Kogi NA-DHS_17	67	0.43	15
Colombia North Wiwa El Encanto		0.43	52
Colombia Zenu NA-DHS_18	75	0.42	16
New Zealand Maori with Full Ancestry	65	0.38	46
Japan Central		0.38	371
Mexico Chihuahua Tarahumara		0.38	44
Colombia Inga NA-DHS_11	53	0.37	16
Japan pop 16		0.36	18604
Japan pop 3		0.36	1018
Costa Rica Guaymi NA-DHS_10	72	0.36	18
USA Arizona Pima		0.36	100
Chile Easter Island		0.36	21
USA Arizona Gila River Pima		0.36	3000
USA NMDP Japanese		0.35	24582
Costa Rica Amerindians	57	0.35	125
USA Hawaii Okinawa		0.34	106
Costa Rica Cabecar NA-DHS_9	53	0.34	19
USA Arizona Gila River Amerindian		0.34	492
Taiwan Pazeh	58	0.34	55
Japan Okinawa Ryukyuan		0.34	143
New Zealand Polynesians with Admixed History	59	0.33	27
American Samoa		0.33	51
Japan pop 5		0.33	117
Philippines Ivatan	58	0.32	50
Papua New Guinea East New Britain Rabaul		0.32	60
New Zealand Polynesians with Full Ancestry	57	0.31	21
USA New Mexico Canoncito Navajo		0.31	42
Australia Yuendumu Aborigine		0.30	191
Australia Groote Eylandt Aborigine		0.29	75
New Zealand Maori with Admixed History	51	0.29	105
9 other populations with HLA-A*24:02 frequencies between 0.24 and 0.29 (not shown)			
Japan Hokkaido Ainu		0.24	50

Data, and also the nomenclature, were retrieved from the Allele Frequency Net Database. Only populations with HLA-A*24:02 frequencies ≥0.29 and Japanese Ainu are listed.

Table 2 lists the subset of the 9-mers that are identical between SARS-CoV-2 and at least one of the CCCoVs ( Dijkstra & Hashimoto, 2020) and additionally were predicted to bind representative MHC-I alleles or experimentally found to bind MHC-I, be part of MHC-II binding peptides, or to activate T cells. It also lists >9-mers for which evidence of T cell activation was reported. The experimental results summarized within Table 2 were collected from the articles listed in Table 1, from articles on SARS-CoV-1, and from reports uniquely deposited to the Immune Epitope Database and Analysis Resource (IEDB; http://www.iedb.org/; Dhanda *et al.*, 2019). The 9-mer peptides listed in Table 2 are referred to in Table 1 by using the letter code of their N-terminal three amino acids.

The only identical T cell epitope repeatedly found to be immunogenic by independent research groups was peptide “VYI” (VYIGDPAQL) (highlighted in bold in Table 1; details in Table 2). The VYI peptide is shared between SARS-CoV-2, HCoV-HKU1, and HCoV-OC43 viruses and part of a larger identical stretch AKHYVYIGDPAQLPAPR in their helicase protein (aka nonstructural protein 13 or NSP13). Prachar *et al.* (2020) showed that the VYI peptide bound to HLA-A*24:02, although not very stable. To our knowledge, all studies that investigated the VYI peptide—three in total—found it to stimulate T cells in an HLA-A*24:02 context ( Ferretti *et al.*, 2020; Kared *et al.*, 2020; Nelde *et al.*, 2021). Ferretti *et al.* (2020) investigated the entire SARS-CoV-2 proteome by a “T-scan” assay measuring activation of CD8 ^+^ memory T cells from COVID-19 convalescent donors after incubation with HEK293 cells engineered to express a single HLA allele and one of a set of overlapping 61 aa stretches. By this method, Ferretti *et al.* (2020) detected only three SARS-CoV-2 stretches with “dominant” epitopes that activated CD8 ^+^ T cells from multiple HLA-A*24:02 ^+^ COVID-19 convalescent donors; one of these three stretches encompassed the VYI peptide and stimulated two of five investigated samples. Involvement of the VYI peptide was confirmed by activation of the HLA-A*24:02 ^+^ T cells upon coculturing with HLA-A*24:02 ^+^ target cells pulsed with VYI peptide. If for the T-scan screen 61 aa stretches of CCCoV proteomes were used instead, Ferretti *et al.* (2020) appear to have found a weak but noticeable response by HLA-A*24:02 ^+^ memory CD8 ^+^ T cells in the cases of HCoV-HKU1 and HCoV-OC43 (our interpretation of their Figure 5A). Kared *et al.* (2020) performed a binding assay, testing 94 peptides from across the SARS-CoV-2 proteome—predicted by software to bind HLA-A*24:02— using pHLA-A*24:02 tetramers for labeling of CD8 ^+^ T cells from five HLA-A*24:02 ^+^ COVID-19 convalescent donors. They found positive reactions for only eight of the 94 peptides. One of these eight peptides was the VIY peptide, which detectably labeled T cells of only one of the five donors. Regarding possible CCCoV-induced anti-SARS-CoV-2 T cell memory, Kared *et al.* (2020) mentioned “Notably, SARS-CoV-2 specific CD8 ^+^ T cells were not detected in any of the healthy donors recruited before the official SARSCoV-2 pandemic;” their number of HD controls, however, was low, and for their HLA-A*24:02-matched experiments seems to have been between one and four. Nelde *et al.* (2021) tested ten predicted HLA-A*24:02 SARS-CoV-2 epitopes and found the VYI peptide to be one of the three “dominant T cell epitopes” as it elicited activation of CD8 ^+^ T cells from seven of ten HLA-A*24:02 ^+^ COVID-19 convalescent donors upon incubation with their PBMC. On the other hand, for PBMC of 17 healthy HLA-A*24:02 ^+^ donors a stimulation of CD8 ^+^ T cells could not be observed. To summarize these three studies, the VYI peptide is among the most immunogenic SARS-CoV-2 T cell epitopes in HLA-A*24:02 ^+^ individuals, although there is no evidence yet that this was primed by previous CCCoV infections. Presumably, because of the latter, none of the three studies mentioned that VYI peptide is shared between SARS-CoV-2, HCoV-HKU1, and HCoV-OC43 ( Ferretti *et al.*, 2020; Kared *et al.*, 2020; Nelde *et al.*, 2021). We assume that not finding anti-VYI T cells in HLA-A*24:02 ^+^ HD was only a matter of assay sensitivity, because CCCoV-induced T cell memory is expected as the VYI peptide is embedded in a longer identical AKHYVYIGDPAQLPAPR stretch shared between SARS-CoV-2, HCoV-HKU1, and HCoV-OC43; thus, the MHC-I pathway processing of the peptide is expected to be similar in each viral background, and software predicts that the immunoproteasome efficiently generates the VYI peptide from all three viruses ( https://imed.med.ucm.es/Tools/pcps/; Gomez-Perosanz *et al.*, 2020). An additional reason for assuming the involvement of CCCoV-induced T cell memory is that the helicase is not one of the more abundant (structural) viral proteins ( Davidson *et al.*, 2020) whereas nevertheless VYI is consistently found as one of the dominant T cell epitopes. Future experiments selectively investigating HLA-A*24:02 donors with a recent HCoV-HKU1 or HCoV-OC43 infection will probably be more sensitive in detecting anti-VYI CD8 ^+^ T cells primed by CCCoV infection.

HLA-A*23:01 belongs to the same “supertype” as HLA-A*24:02 ( Lund *et al.*, 2004) meaning that they tend to bind similar peptides, and in the IEDB database it is reported that HLA-A*23:01 also binds VYI peptide (IEDB Reference:1000425). It has not been described yet whether VYI peptide can stimulate T cells in an HLA-A*23:01 context.

Although not identical throughout the sequence, as an exception, Table 2 also lists the 9-mer SPRWYFYYL (“SPR”) for SARS-CoV-2 and its matching LPRWYFYYL (“LPR”) for HCoV-HKU1 and HCoV-OC43. Several independent studies ( Table 1; SPR indicated in bold) suggest that SPR is highly immunogenic and involved in cross-virus immune memory (summarized in Table 2). The only amino acid difference between the SPR and LPR peptides is at the P1 position, which is not necessarily important for peptide conformation in pMHC-I complexes or for binding T cell receptors (TCRs) (e.g., Sewell, 2012). Thus, it seems plausible that a fraction of the T cells primed by pMHC/LPRWYFYYL may also recognize pMHC/SPRWYFYYL. It was convincingly shown that SPR peptide binds HLA-B*07:02 alleles and in that context can stimulate CD8 ^+^ T cells from COVID-19 convalescent donors ( Ferretti *et al.*, 2020; Kared *et al.*, 2020; Peng *et al.*, 2020; Schulien *et al.*, 2021; Snyder *et al.*, 2020). CD8 ^+^ T cells from HLA-B*07:02 ^+^ HD could also be stimulated by SPR, suggesting cross-virus CD8 ^+^ T cell memory induced by the LPR peptide of HCoV-HKU1 or HCoV-OC43 ( Schulien *et al.*, 2021), which is consistent with *in vitro* results showing that CD8 ^+^ memory T cells from HLA-B*07:02 ^+^ COVID-19 convalescent donors were activated by HLA-matched cells expressing 61 aa fragments of HCoV-HKU1 or HCoV-OC43 encompassing the LPR peptide ( Ferretti *et al.*, 2020). However, SARS-CoV-2 peptides encompassing or even only partially overlapping the SPR peptide also induced responses—remarkably strong in some cases—of CD4 ^+^ T cells from COVID-19 convalescent donors and, to a lesser extent, from HD (also summarized in Table 2; Le Bert *et al.*, 2020; Nelde *et al.*, 2021; Peng *et al.*, 2020; Tarke *et al.*, 2020). The combined results suggest an overlap of highly immunogenic MHC-I and MHC-II epitopes, and—although such overlap is not impossible—some caution for the possibility that SPR peptide might (additionally) cause non-MHC-restricted immune stimulation seems to be warranted. Except for HLA-B*07:02, SPR peptide was also found to bind the MHC-I alleles HLA-B*51:01, -*53:01, and -*54:01 (IEDB database Reference:1000425).

Other than VYI, we did not find any other identical peptides shared between SARS-CoV-2 and CCCoVs for which current publications convincingly indicate a high immunogenicity. However, the 9-mers FVDGVPFVV (“FVD”) and LPYPDPSRI (“LPY”) could be promising, although they were only reported as immunogenic CD8 ^+^ T cell epitopes in single publications. Snyder *et al.* (2020) appear to have identified FVD peptide as an immunodominant SARS-CoV-2 T cell epitope in the HLA-A*02:01 context, although their descriptions of this matter could be more detailed. Tarke *et al.* (2020) found that CD8 ^+^ T cells from a few HLA-B*51:01 ^+^ COVID-19 convalescent donors could be stimulated by several peptides that encompassed the LPY 9-mer peptide sequence, namely peptide DYVYLPYPDPSRI (a 13-mer shared between SARS-CoV-2 and HCoV-HKU1), its shorter versions VYLPYPDPSRI (an 11-mer) or YLPYPDPSRI (a 10-mer), or peptide LPYPDPSRIL (a 10-mer); software predicts that the encompassed LPYPDPSRI (a 9-mer) is the best HLA-B*51:01 binder and that of the other lengths only the 10-mers YLPYPDPSRI and LPYPDPSRIL are expected to bind this allele ( Table 2), suggesting that some processing of the longer peptides may explain the combined results. Future analysis of the immunogenicity of the LPY 9-mer peptide would be interesting.

In addition to the above, Mateus *et al.* (2020) and Tarke *et al.* (2020) found some other SARS-CoV-2 peptides that encompassed ≥9-mers shared with CCCoVs and stimulated CD4 ^+^ T cells ( Table 2), but whether those identical stretches formed the immunogenic epitope has not been determined yet.

### Approximately 60% of the Japanese population carries the MHC class I allele HLA-A*24:02

**Figure 3.**
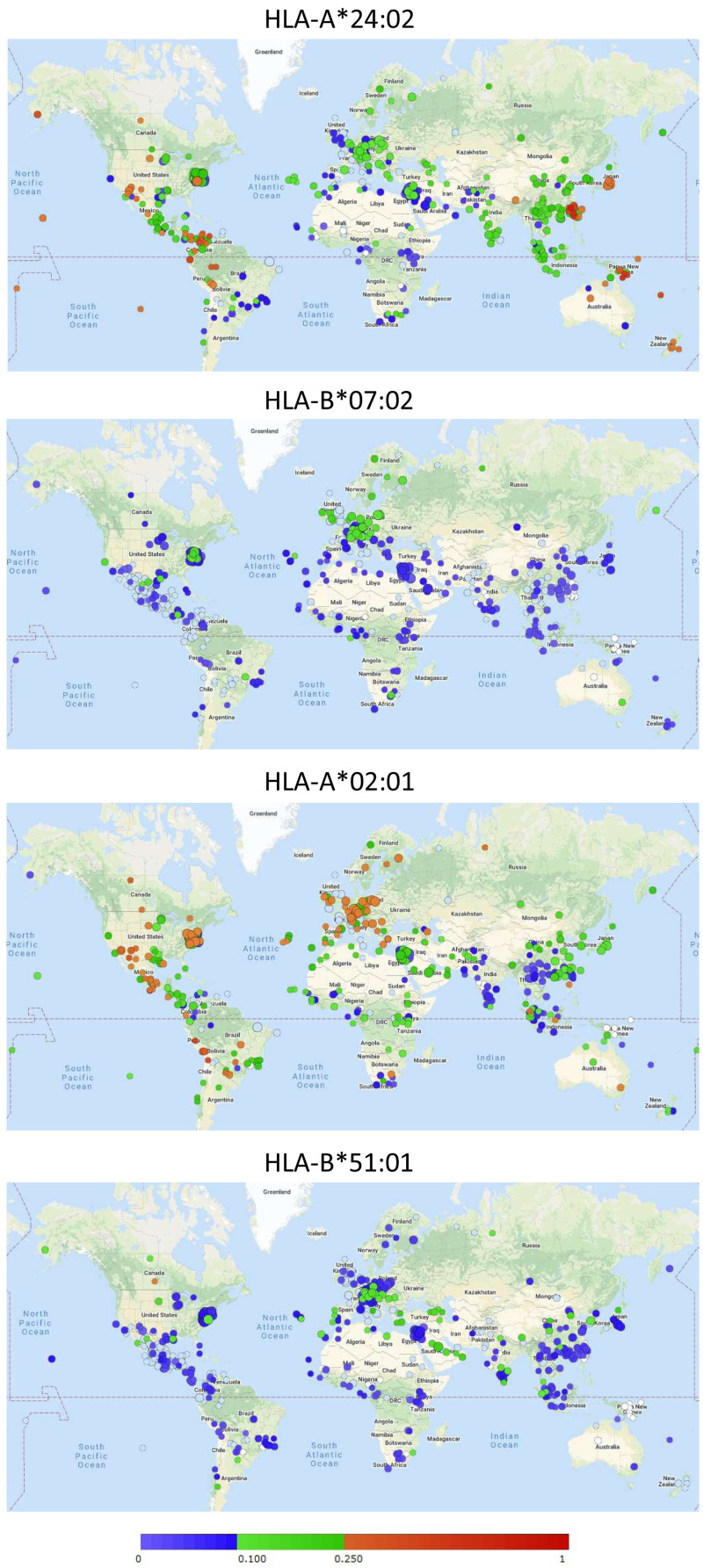
Global distribution of HLA-A*24:02, HLA-B*07:02, HLA-A*02:01, and HLA-B*51:01 allele frequencies as visually summarized by the Allele Frequency Net Database ( Gonzalez-Galarza *et al.*, 2020). Circles refer to individual studies with the color indicating the detected allele frequency following the color bar. See http://www.allelefrequencies.net/ for more detailed information on those studies. Permission to reproduce this image was obtained from AFND.

For speculation on the global implications of the ability of different MHC-I alleles to bind conserved peptides, their global distributions must be appreciated. The online Allele Frequency Net Database (AFND; http://allelefrequencies.net/; Gonzalez-Galarza *et al.*, 2020) comprises information of MHC-I allele frequencies in different populations, and Figure 3 includes their visual summaries of global distributions of the alleles HLA-A*24:02 (binder of VYI peptide), HLA-B*07:02 (binder of SPR peptide), HLA-A*02:01 (binder of FVD peptide), and HLA-B*51:01 (predicted binder of LPY peptide). Table 3 lists populations with the highest frequencies of HLA-A*24:02 according to AFND. For all studies summarized in Table 3 the allele frequencies and for a subset also the percentage of individuals carrying the allele had been determined (note that if randomly distributed, an allele frequency of >0.29 would correspond to a prevalence of >50%). Data for populations in Japan and for Japanese in the USA are highlighted in shades of gray ( Table 3). In Japanese populations the HLA-A*24:02 allele frequencies are ≥0.33 except for in the Ainu which are a racial minority indigenous to Hokkaido in Northern Japan. Ikeda *et al.* (2015; the “Japan pop 16” study in Table 3) investigated 18604 individuals from “all parts of Japan” and the authors concluded that the HLA-A*24:02 allele is distributed in ~60% of the Japanese population. Other populations in which HLA-A*24:02 is present in >50% of the investigated members are various indigenous populations in Asia, Oceania, and the Americas ( Table 3). Those non-Japanese populations are not further discussed in this study as it is harder to collect their data relevant to COVID-19.

HLA-B*07:02 is common in Northern Europe ( Figure 3) and is carried by approximately a third of the Irish population (reports at AFND). HLA-A*02:01 is common in Europe, and, for example, found in 49% of the Polish population (report at AFND). HLA-B*51:01 is common in Southern Europe and the Middle East ( Figure 3) and was found at an allele frequency of 0.19 in Saudi Arabia (report at AFND).

### COVID-19 and CCCoV infections in Japan

A year into the pandemic, the accumulated number of COVID-19 deaths per million inhabitants in Japan is 45, which is >30 times fewer than in countries such as Italy (1465), the UK (1559), and the USA (1362) (on February 1st, 2021, according to https://www.worldometers.info/coronavirus/). At least for the initial wave of the disease in the first half of 2020 the apparent low prevalence of COVID-19 was supported by finding specific antibodies in only ~0.1% of citizens of Tokyo in June 2020 (governmental report https://www.mhlw.go.jp/content/000648706.pdf) and minimal or even negative excess mortality rates until at least July 2020 ( Yorifuji *et al.*, 2021). These low numbers are quite surprising since the stringency of behavioral regulations to protect against COVID-19 have been less severe in Japan than in most Western countries ( https://www.bsg.ox.ac.uk/research/research-projects/coronavirus-government-response-tracker; Hale *et al.*, 2020). The surprise about the relatively low incidence of COVID-19 in Japan was captured well in the title of a BBC article on July 2020 “Coronavirus: Japan's mysteriously low virus death rate” ( https://www.bbc.com/news/world-asia-53188847). Most of the difference with Western countries can probably be explained by voluntary behaviors such as the willingness of the Japanese to wear masks (despite absence of obligation) and by better individual health status such as a relatively low prevalence of obesity. As a note, however, the Japanese are not per se better protected against respiratory viruses, as mortalities per capita resulting from the 2009 influenza pandemic were higher than in European countries ( Simonsen *et al.*, 2013).

In Japan, as in other countries, CCCoV infections are poorly monitored, but available data indicate that from the winter 2014–2015 until November 2019 (we were not able to find more recent data) the most common CCCoV species in Japan has been HCoV-OC43. Especially during the winters of 2014–2015 and 2018–2019, this virus species appears to have been prevalent ( Komabayashi *et al.*, 2020; Kubota-Koketsu *et al.*, 2020). Thus, many Japanese individuals probably received a relatively recent immune boost with the VYI peptide.

Currently, in the winter 2020–2021, there has been a surge in the number of COVID-19 cases and deaths in Japan ( https://www.worldometers.info/coronavirus/; https://www.aljazeera.com/news/2021/1/4/japan-weighs-state-of-emergency-amid-severe-covid-19-surge), and it is unclear whether the factors that during the first half of 2020 protected the Japanese better than the populations of many other countries are still in place. Possibly, the warm winter season of 2019–2020 (Japan Meteorological Agency reports https://www.data.jma.go.jp/obd/stats/data/en/smp/index.html), and the associated early hay fever season ( https://global.weathernews.com/news/13178/; http://kafun.taiki.go.jp/), may have helped to protect the Japanese population during the first half of 2020. In Japan, largely because of aging monoculture coniferous forests, depending on the definition approximately half of the population may be considered to suffer from pollen-induced allergic rhinitis, which has been called a national affliction (e.g., Minami *et al.*, 2019; Nakamura *et al.*, 2019; Yamada *et al.*, 2014). Although speculative, the associated inflammation of the respiratory tract might non-specifically elevate both innate and specific (possibly anti-VYI T cells) immunity against COVID-19. Although each of these individual biological factors probably is not very protective against COVID-19 (see also the below paragraph), at the population scale a combination of such factors might significantly impact the virus reproduction number (R).

In short, (i) most Japanese individuals possess the MHC-I allele HLA-A*24:02 that presents a highly immunogenic SARS-CoV-2 T cell epitope VYI, (ii) in recent years many of them have been exposed to this epitope by HCoV-OC43 infection, and (iii) at the time of the first COVID-19 wave many of them had an elevated immune status of their respiratory tracts because of pollen allergy.

### How might MHC polymorphism affect anti-COVID-19 immunity?

MHC polymorphism is believed to be driven by differences in immune responses conferred by the different alleles, but actual evidence for this to cause differences in disease resistance is close to absent ( Kelly & Trowsdale, 2017; Yamaguchi & Dijkstra, 2019). Presumably, this is caused by each set of MHC alleles having enough choice within a pathogen proteome for presenting some peptides efficiently to the immune system. Theoretically, the MHC allelic effect on differences in disease resistance should become larger if the choice of possible immunogenic epitopes becomes more limited. Hence, the effect of MHC polymorphism on immune memory induced against a related virus should be larger than on immune memory against the same virus. However, several studies have investigated the association of MHC polymorphism with differences in COVID-19 resistance, and—arguably—no convincing associations have yet been presented ( Iturrieta-Zuazo *et al.*, 2020; Littera *et al.*, 2020; Lorente *et al.*, 2021; Wang *et al.*, 2020). From these combined within population studies, however, it probably follows that HLA-A*24:02 does not necessarily have a detectable positive or negative impact on the two commonly analyzed COVID-19 parameters, which are the frequency of contracting COVID-19 and the severity of the disease. As far as we know, there has not been a thorough investigation yet of a possible association between MHC polymorphism and the virus titers in the upper respiratory tract. The severity of COVID-19 is predominantly determined by whether the virus infects the lower respiratory tract ( Cyranoski, 2020), which, curiously, has been described as largely disconnected from the level of virus replication in the upper respiratory tract ( He *et al.*, 2020; Lavezzo *et al.*, 2020). He *et al.* (2020), for example, stated “There was no obvious difference in viral loads across sex, age groups and disease severity.” Therefore, if cross-virus memory T cells would not be sufficient to help block an infection but instead help to expedite the end of upper respiratory tract infections, the HLA-A*24:02 allele might give protection at the population level by limiting spread without having an impact on the COVID-19 parameters typically studied in the HLA-association studies (contraction and severity at the level of individuals). We speculate that HLA-*24:02-restricted anti-VYI CD8+ T cell immune memory—especially if recently boosted by HCoV-OC43 or HCoV-HKU1 infections or nonspecific immune stimulations—can reduce the total number of virus particles secreted by COVID-19 patients and so the replication number (R) of the virus at the population level.

## Concluding remarks

The only CD8 ^+^ T cell epitope shared between CCCoVs and SARS-CoV-2 for which the immunogenicity was convincingly proven is the VYIGDPAQL peptide if presented by HLA-A*24:02. This MHC-I allele is found in the majority of the Japanese population and may help explain their surprising resistance to the virus. More studies on T cells activated during CCCoV infections and on possible associations of MHC alleles with COVID-19 parameters are necessary. Considering that for the S protein there may not be meaningful CCCoV-induced anti-SARS-CoV-2 cross-virus immune memory (depending on how one interprets the various publications)—related to the weak conservative pressure on this protein—it is feasible that SARS-CoV-2 will mutate its S proteins and escape the immune protection induced by the current generation of S-only vaccines. Potentiating such vaccines by adding immunogenic peptides from better conserved parts of the proteome, for example VYI peptide in the case of HLA-A*24:02 ^+^ individuals, may be a viable option to help prevent such immune escape.

## Data availability

Severe acute respiratory syndrome coronavirus 2 isolate Wuhan-Hu-1, complete genome, Accession number MN908947: https://www.ncbi.nlm.nih.gov/nuccore/MN908947

Human coronavirus OC43, complete genome, Accession number NC_005147.1: https://www.ncbi.nlm.nih.gov/nuccore/NC_005147.1

Human coronavirus HKU1, complete genome, Accession number NC_006577: https://www.ncbi.nlm.nih.gov/nuccore/NC_006577

Human coronavirus 229E, complete genome, Accession number NC_002645: https://www.ncbi.nlm.nih.gov/nuccore/NC_002645

Human Coronavirus NL63, complete genome, Accession number NC_005831: https://www.ncbi.nlm.nih.gov/nuccore/NC_005831
