# Supplementary material for: Most Japanese individuals are genetically predisposed to recognize an immunogenic protein fragment shared between COVID-19 and common cold coronaviruses
Source: F1000Res. 2021 Mar 10;10:196. [Version 1] doi: 10.12688/f1000research.51479.1 (PMC8108557; doi:10.12688/f1000research.51479.1)
Supplement: Peer review history

# Peer review: Reviewer response for version 1

**DOI:** 10.5256/f1000research.54653.r81415

**Published:** 2021-05-05

**Authors:** Anna Gil

**Affiliations:** 1 Department of Pathology, University of Massachusetts Medical School, Worcester, MA, USA

This manuscript summarizes a very important report on possible MHC-I binding epitopes shared between common cold coronaviruses and SARS-CoV-2. It presents a detailed and thorough analysis of existing literature in a single setting. Based on the reports focused on T cell response restricted to HLA-A24:02, the authors rightly suggest that the future vaccine design should include immunogenic peptides from more conserved parts of the SARS-CoV2 proteome.  

Is the work clearly and accurately presented and does it cite the current literature?

Yes

If applicable, is the statistical analysis and its interpretation appropriate?

I cannot comment. A qualified statistician is required.

Are all the source data underlying the results available to ensure full reproducibility?

Yes

Is the study design appropriate and is the work technically sound?

Yes

Are the conclusions drawn adequately supported by the results?

Yes

Are sufficient details of methods and analysis provided to allow replication by others?

Yes

Reviewer Expertise:

T cell immunology in humans, T cell receptor repertoire, CD8+T cell cross-reactivity

I confirm that I have read this submission and believe that I have an appropriate level of expertise to confirm that it is of an acceptable scientific standard.

# Peer review: Reviewer response for version 1

**DOI:** 10.5256/f1000research.54653.r81412

**Published:** 2021-04-26

**Authors:** Andrea J. Sant

**Affiliations:** 1 David H. Smith Center for Vaccine Biology and Immunology, University of Rochester Medical Center, Rochester, NY, USA

This manuscript describes the process of definitive epitope discovery for SARS-Cov-2 and the relevance of particular peptide epitopes because of the prevalence of particular HLA restriction elements in particular populations, and the potential for cross-reactive memory to be recalled from immunity established from endemic seasonal coronavirus infections. Thus, the impact of definitive epitope discovery is high in these situations.

 While the conclusions of the study and the major strategies offered are valuable, some trimming of the manuscript could make this more accessible to the reader. I recommend that the authors select the most essential features of past studies to illustrate key points that are necessary to present to the reader.

 I also recommend that the peptide be named by its full spanning amino acids (first and last) for clarity rather than “VYI”.

Is the work clearly and accurately presented and does it cite the current literature?

Yes

If applicable, is the statistical analysis and its interpretation appropriate?

I cannot comment. A qualified statistician is required.

Are all the source data underlying the results available to ensure full reproducibility?

Yes

Is the study design appropriate and is the work technically sound?

Yes

Are the conclusions drawn adequately supported by the results?

Yes

Are sufficient details of methods and analysis provided to allow replication by others?

Yes

Reviewer Expertise:

CD4 T cells and MHC restricted antigen presentation

I confirm that I have read this submission and believe that I have an appropriate level of expertise to confirm that it is of an acceptable scientific standard, however I have significant reservations, as outlined above.

# Peer review: Reviewer response for version 1

**DOI:** 10.5256/f1000research.54653.r81416

**Published:** 2021-04-26

**Authors:** Brian M. Baker

**Affiliations:** 1 Department of Chemistry and Biochemistry, Harper Cancer Research Institute, University of Notre Dame, Notre Dame, IN, USA

Dijkstra and colleagues describe a meta analysis of potential cross-reactivity between T-cell epitopes of common coronaviruses and SARS-CoV2. After a comprehensive review, they identify only one possible HLA-A2402 presented cross-reactive epitope. As a meta analysis there is no new experimental data provided to review, but the the analysis of prior data is comprehensive and rigorous. The work will be valuable in responding to the ongoing CoV2 pandemic, understanding immunity (and potential pre-existing immunity), and in the planning of next generation vaccines.

Is the work clearly and accurately presented and does it cite the current literature?

Yes

If applicable, is the statistical analysis and its interpretation appropriate?

Yes

Are all the source data underlying the results available to ensure full reproducibility?

Yes

Is the study design appropriate and is the work technically sound?

Yes

Are the conclusions drawn adequately supported by the results?

Yes

Are sufficient details of methods and analysis provided to allow replication by others?

Yes

Reviewer Expertise:

T cell immunology, T cell recognition, antigen presentation, immune crossreactivity.

I confirm that I have read this submission and believe that I have an appropriate level of expertise to confirm that it is of an acceptable scientific standard.
